# Supplementary material for: Ambient PM2.5 and PM10 Exposure and Respiratory Disease Hospitalization in Kandy, Sri Lanka
Source: Int J Environ Res Public Health. 2021 Sep 12;18(18):9617. doi: 10.3390/ijerph18189617 (PMC8466407; doi:10.3390/ijerph18189617)
Supplement: Supplementary file 1 [file ijerph-18-09617-s001.zip › ijerph-1364383-supplementary.pdf]

Supplementary Materials

Figure S1. Kandy District, Sri Lanka (Land area 1910 km^2; Population 1.5 million approx.).

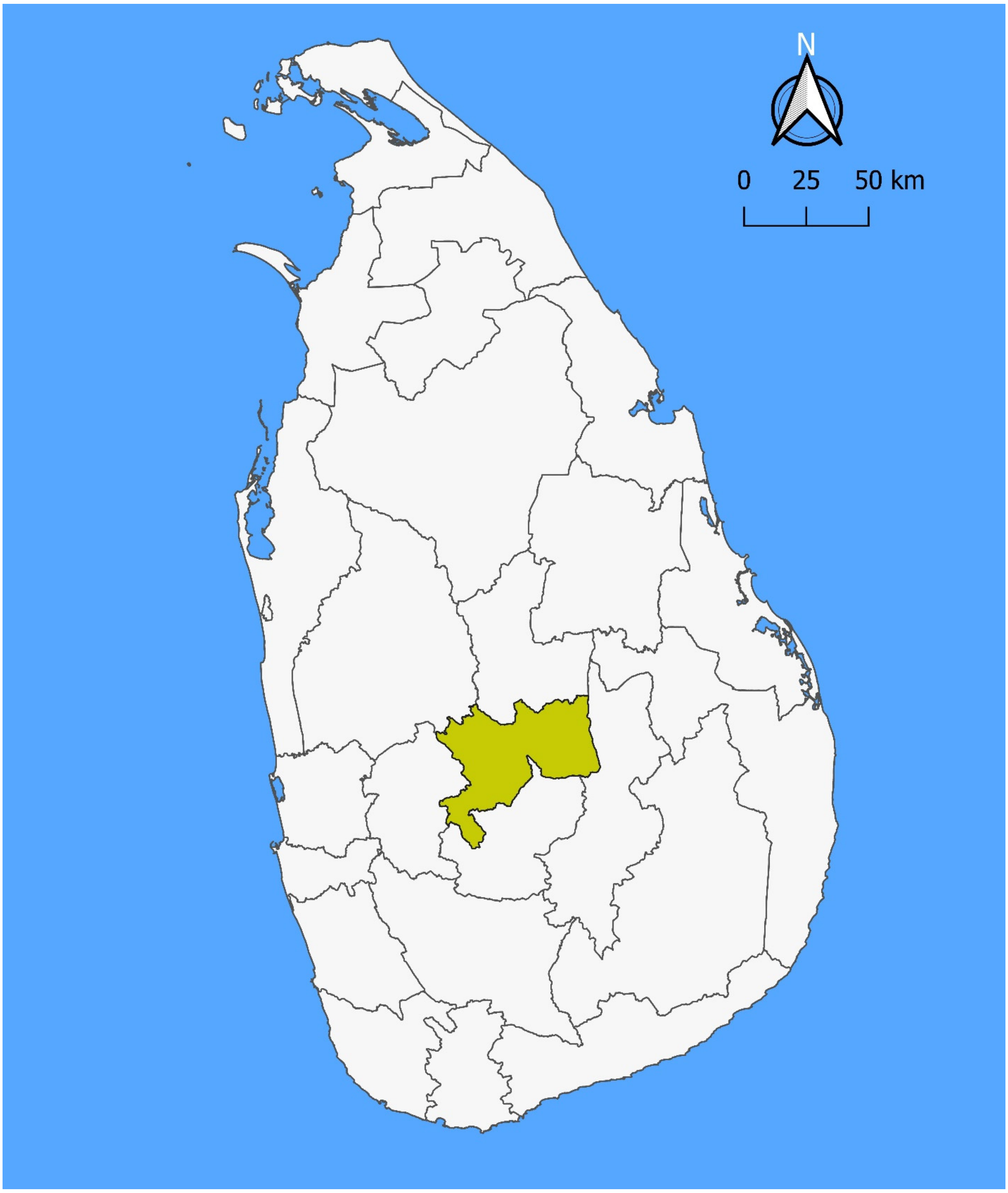

Figure S2: Divisional secretariats of the Kandy District. Catchment area of the Teaching Hospital, Kandy includes regions 0, 4-7, 9, 11-15 Catchment area of the Teaching Hospital, Peradeniya includes regions 1-4, 6, 10, 12-14, 16-18.

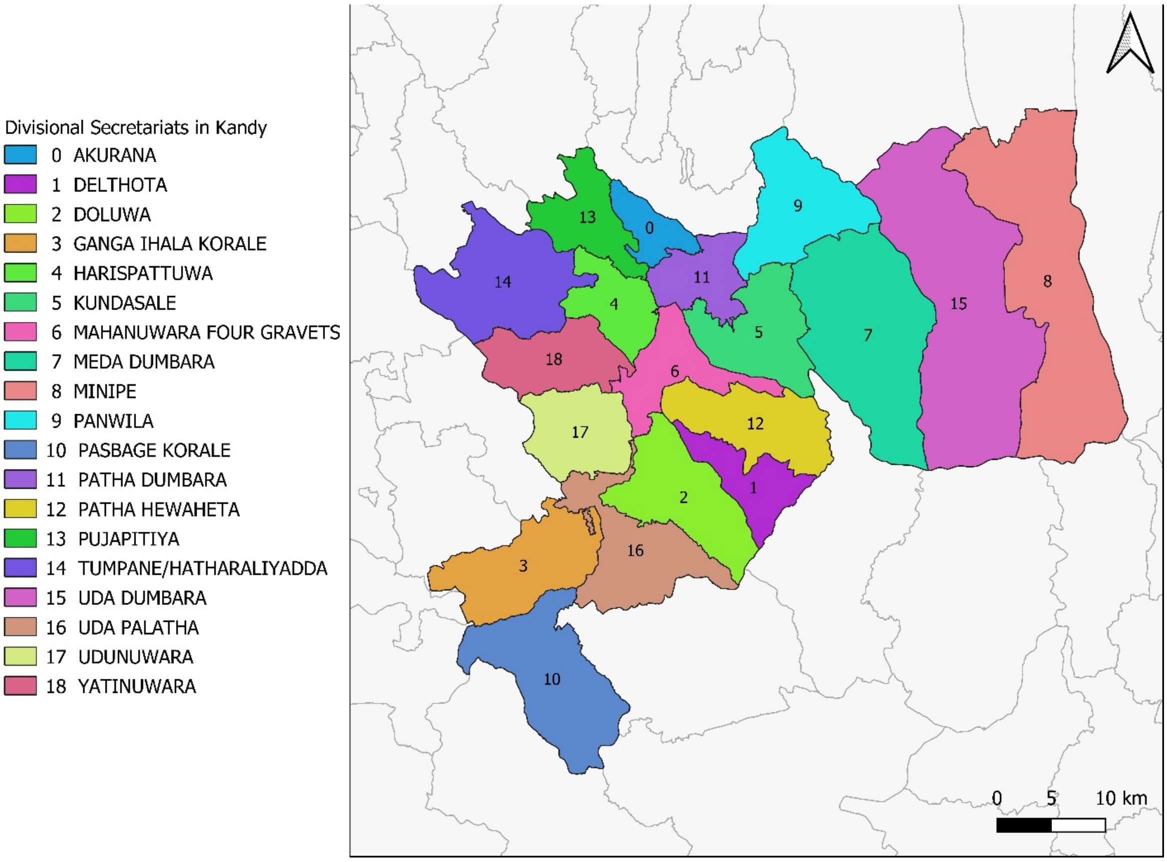

Figure S3. The locations of the air pollution monitoring sites in Kandy in 2020.

(KOALA 82 – National Institute of Fundamental Studies, KOALA 84 - Asgiriya, KOALA 87 - Fac-ulty of Allied Health Sciences University of Peradeniya, KOALA 88 - Keerthi Sri Rajasinghe Mawatha)

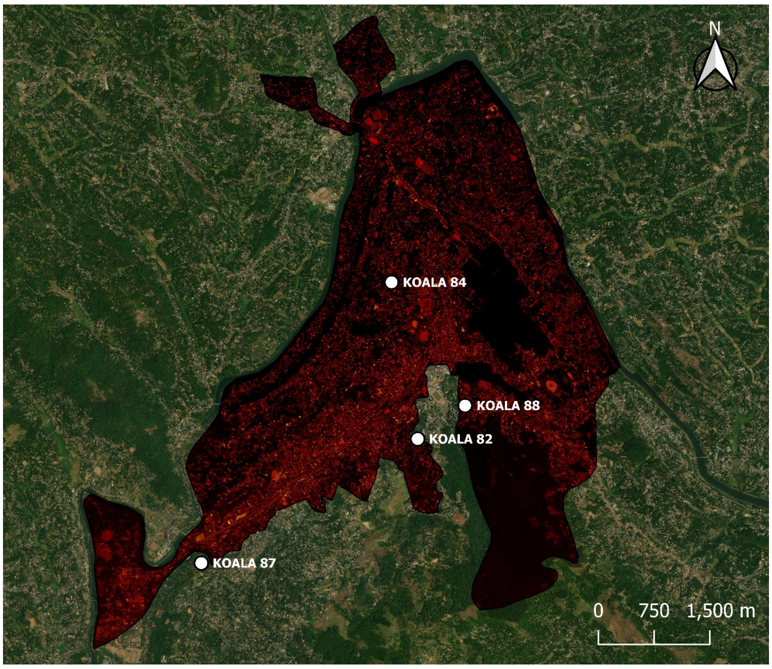

Table S1: *The number of hospitalizations due to respiratory diseases from 01-01-2019 to 12-31-2019*

	Number of hospitalization (%) N=9709
Male	5221 (53.77)
Female	4488 (46.23)
≤64 years	6129 (63.13)
65+ years	3580 (36.87)
Pneumonia	271 (2.79)
COPD	2238 (23.05)
Asthma	1988 (20.48)

Table S2: *The number of hospitalizations due to respiratory diseases in low and high air pollution periods in selected months*

	Low ambient air pollution period	High ambient air pollution period
	Number of hospitalizations (%)	Number of hospitalizations (%)
All	2427 (45.17)	2946 (54.83)
Male	1262 (23.49)	1628 (30.30)
Female	1165 (21.68)	1318 (24.53)
0–64 years	1586 (29.52)	1846 (34.36)
65+ years	841 (15.65)	1100 (20.47)
Pneumonia	58 (1.08)	90 (1.68)
COPD	525 (9.77)	707 (13.16)
Asthma	534 (9.94)	559 (10.40)

Table S3: *Descriptive statistics for daily air pollutants in Kandy, 2020 measured at different locations in Kandy.*

KOALA ID	Time Period	$PM_{2.5}$	PM_{10}
KOALA82 (NIFS)	Feb-01 to Mar-02	37.21 (9.73)	44.03 (11.59)
KOALA84	Feb-08 to Mar-29	40.27 (7.51)	45.39 (8.57)
KOALA87	Feb -11 to Mar -01	36.93 (9.97)	40.74 (11.31)
KOALA88	Feb -01 to Mar-01	37.59 (7.77)	42.99 (9.14)
